# Uterine Fundectomy in Patients With Benign Etiology Undergoing Hysterectomy: New Surgical Technique

**DOI:** 10.2196/resprot.7536

**Published:** 2017-10-10

**Authors:** AboTaleb Saremi, Homa Bahrami, Fariba Feizy

**Affiliations:** ^1^ Sarem Fertility & Infertility Research Center (SAFIR) Tehran Islamic Republic Of Iran; ^2^ Sarem Cell Research Center (SCRC) Tehran Islamic Republic Of Iran; ^3^ Sarem Women’s Hospital Tehran Islamic Republic Of Iran

**Keywords:** hysterectomy, uterine, fundectomy

## Abstract

**Background:**

Hysterectomy is the most common surgical procedure in gynecology, not only in cases of malignancies but also in many benign cases. Many uterine preservation techniques have been introduced as alternatives to hysterectomy.

**Objective:**

We aimed to propose a new uterine surgical procedure. In this paper, we compare the utility of this new technique to the limitations of current procedures.

**Methods:**

Uterine fundectomy may be considered as a subtotal hysterectomy. In this new technique, the uterine fundus including all pathologic tissue is cut as a reverse trapezoid by monopolar cautery. The upper side of the trapezoid, which includes the whole uterine fundus, is removed, but the fallopian tubes and cornual segment are preserved. A small uterine cavity remains, as well as the endometrial tissue lining it.

**Results:**

Patient recruitment for this study began in April 2017 and is expected to end approximately 12 months later. Assessment of the primary outcomes is expected to take place in April 2018.

**Conclusions:**

Uterine preservation is particularly critical in developing new surgical approaches that can lead to a positive impact on patient satisfaction. This protocol outlines the first attempt to prospectively test surgical fundectomy in candidates for hysterectomy for benign indications.

## Introduction

Hysterectomy is the most common surgical procedure in gynecology, not only in cases of malignancies but also in many benign cases, such as uterine fibroids, endometrial hyperplasia, adenomyosis, dysfunctional uterine bleeding, and cervical intraepithelial neoplasia [1]. For benign cases, there are many possible hysterectomy procedures. The surgical technique is selected based on the reason for surgery, the general condition of the patient, and the surgeon’s experience and skills. A variety of surgical techniques exists, including minimally invasive procedures (laparoscopic) and traditional open surgical procedures (laparotomy). Minimally invasive surgical procedures include laparoscopic hysterectomy and robotic-assisted laparoscopic hysterectomy [2]. Open surgical procedures include radical hysterectomy, where surgeons remove the uterus and surrounding tissues and cervix; a total hysterectomy, where the entire uterus and cervix are removed; and subtotal or partial hysterectomy, where the uterus is surgically removed but the cervix is left in place.

In the past decade, many uterine preservation techniques have been introduced as alternatives to hysterectomy. These techniques use modern technology and show reliable and comparable results [3]. In a randomized controlled trial, women in the subtotal hysterectomy group had a significantly better quality of life and improved body image compared to the total hysterectomy group [4]. It seems that hysterectomy performed with ovary preservation can still affect the function of the ovaries. It has been reported in women who have undergone hysterectomy compared with the control group (no surgical procedure) that postmenopausal symptoms occur earlier, probably due to the reduction of ovarian blood flow.

Here we aim to propose a new uterine surgical procedure and to compare the utility of this technique to the limitations of present procedures.

## Methods

### Overview

Uterine fundectomy is used only in patients who have indications for benign uterine conditions, where only the pathologic part of the uterine body is removed. The neck of the uterus (cervix) and a part of the uterine and endometrial tissue are preserved, so that menstrual periods remain. An important feature of uterine fundectomy is the preservation of the uterus in women after surgical treatment. Thus, uterine blood vessels, adnexa (appendages), and ovaries remain with no surgical manipulation and ovarian function is not affected. The most important thing about this technique is the choice of patient. This procedure is performed only in benign cases of patients with uterine surgical indication, and in those who wish to preserve the uterus. However, this procedure does not preserve fertility because the remaining uterine cavity is very small. Replacing a hysterectomy with the uterine fundectomy in appropriate cases can preserve menstrual bleeding and ovarian function, as well as maintaining a positive impact on patient satisfaction.

### Recruitment

Patients will be initially recruited from a pool of women who choose to have a hysterectomy for benign indications. Researchers will then notify them about the study protocol and ask about their interest in participating. Inclusion criteria consists of premenopausal women aged 36-50 years, candidates for hysterectomy for benign indications, and those patients who signed the informed consent and agree to participate. Women with a history of cancer, hereditary cancer, and those patients who are candidates for surgery because of a malignant pathology will be excluded.

### Surgical Procedure

**Figure 1:**
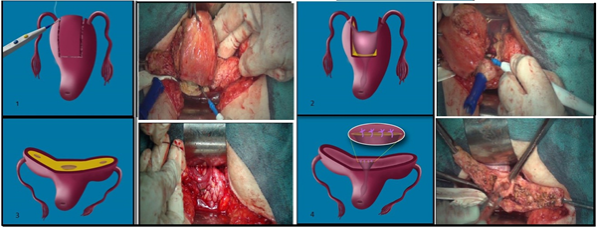
Illustration and photos of the new uterine surgical procedure.

In uterine fundectomy, to prevent intra-operative bleeding, a tourniquet is placed on the lower segment of the uterus below the ovaries. Thus, uterine and ovarian arteries are temporarily closed. Then, the uterine body is cut as a reverse trapezoid by monopolar cautery (Figure 1). The upper side of the trapezoid, which includes the whole uterine fundus, is removed, but the fallopian tubes and cornual segment are preserved. The lower and smaller border of the trapezoid is 1 cm above the internal os of the uterus. Thus, a small uterine cavity remains, as well as the endometrial tissue lining it. The roof of the new uterine cavity, is closed separately by a 2-0 vicryl suture. The lateral uterine segments are sutured to the upper surface and closure is performed. Dead space closure is performed with 0 vicryl suture. After ensuring complete restoration, the tourniquet is opened and complete homeostasis established.

### Outcomes

Primary outcome measures will be the number of participants having subjective symptoms after fundectomy. Sexual symptoms will be assessed with a questionnaire. Secondary outcome measures will be the evaluation of pelvic organ prolapse after surgery and patients’ quality of life.

### Ethical Approval

Women will be asked to sign the informed consent after reviewing the study protocol and consent form. The research will be approved by Sarem Hospital Ethical Committee.

## Results

Patient recruitment began in April 2017 and is expected to end approximately 12 months later. Thus, assessment of primary outcomes of interest is expected in April 2018.

## Discussion

### Principal Findings

Uterine preservation is particularly critical in development of new surgical approaches that can lead to a positive impact on patient satisfaction. In the current subtotal hysterectomy, uterine arteries are cut off and thus the blood supply and ovarian adnexal flow are diminished, ultimately affecting ovarian function. This almost certainly affects the patients’ quality of life. But in uterine fundectomy, because there is no manipulation of the uterine arteries, typically no ovarian dysfunction occurs. In addition, in uterine fundectomy no harm occurs to the pelvic floor, minimizing subsequent issues. The duration of surgery and the amount of bleeding in uterine fundectomy are less than in the other techniques. Further, uterine preservation may also have a psychologically positive effect on a woman’s quality of life. However, it should be noted that uterine fundectomy does not preserve fertility, because the remaining uterine cavity is very small and the amount of endometrial lining is minimal.

### Conclusion

This protocol outlines the first attempt to prospectively test surgical fundectomy in patients who are candidates for hysterectomy for benign indications. Study results are expected to show improved quality of life in patients, offering more positive options for women in the future.
