# Metadata Correction: Uterine Fundectomy in Patients with Benign Etiology Undergoing Hysterectomy: New Surgical Technique

**DOI:** 10.2196/10751

**Published:** 2018-06-28

**Authors:** AboTaleb Saremi, Homa Bahrami, Fariba Feizy

**Affiliations:** ^1^ Sarem Fertility & Infertility Research Center (SAFIR) Tehran Islamic Republic Of Iran; ^2^ Sarem Cell Research Center (SCRC) Tehran Islamic Republic Of Iran; ^3^ Sarem Women’s Hospital Tehran Islamic Republic Of Iran

The authors of the paper “Uterine Fundectomy in Patients with Benign Etiology Undergoing Hysterectomy: New Surgical Technique” (JMIR Res Protoc 2017;6(19):e150) regret that an error occurred in the listing of some affiliation addresses in their original paper and would like to apologize for any inconvenience caused.

The correct listings are as follows:

^1^Sarem Fertility and Infertility Research Center (SAFIR), Tehran, Islamic Republic of Iran

^2^Sarem Cell Research Center (SCRC), Tehran, Islamic Republic of Iran

^3^Sarem Women’s Hospital, Tehran, Islamic Republic of Iran

The corrected article will appear in the online version of the paper on the JMIR website on June 28, 2018, together with the publication of this correction notice. Because this was made after submission to PubMed, Pubmed Central, and other full-text repositories, the corrected article also has been re-submitted to those repositories.

